# Osteonecrosis of the Jaw Associated with Antiangiogenics in Antiresorptive-Naïve Patient: A Comprehensive Review of the Literature

**DOI:** 10.1155/2018/8071579

**Published:** 2018-04-23

**Authors:** Kununya Pimolbutr, Stephen Porter, Stefano Fedele

**Affiliations:** ^1^UCL Eastman Dental Institute, London, UK; ^2^Department of Oral Medicine and Periodontology, Faculty of Dentistry, Mahidol University, Bangkok, Thailand; ^3^NIHR University College London Hospitals Biomedical Research Centre, London, UK

## Abstract

**Objectives:**

To review the available literature on medication-related osteonecrosis of the jaw (MRONJ) associated with antiangiogenics in antiresorptive-naïve individuals.

**Methods:**

A literature search was performed using MEDLINE via PubMed, EMBASE, and Web of Science in December 2017.

**Results:**

We identified reports describing a total of 35 antiresorptive drugs-naïve patients who developed antiangiogenic-related MRONJ. The mean age of these patients was 59.06 years and the F : M ratio was 4 : 5. The most common underlying disease was metastatic renal cell cancer. Pain to the mandible was the most common complaint (34.29%) and the majority of patients presented with bone exposure. The mean duration of intravenous and oral antiangiogenics before MRONJ development was 6.5 and 16.72 months, respectively. The most common additional risk factor was dental extraction (37.14%). Almost half of the MRONJ patients (48.57%) received surgical treatment. 18 patients (62.06%) were reported to have disease resolution within an average time of 6.75 months.

**Conclusion:**

MRONJ associated with antiangiogenic therapy in antiresorptive-naïve patients is a rare but potentially serious adverse effect. Available data suggests that there might be notable differences between MRONJ associated with antiangiogenics and antiresorptives; however, further prospective well-designed studies are required.

## 1. Introduction

Medication-related osteonecrosis of the jaw (MRONJ) is an uncommon and potentially serious adverse side effect of antiresorptive and antiangiogenic agents [1]. It can cause chronic pain, infection, dysfunction, and disfigurement and can affect the quality of life of affected individuals [2, 3]. The vast majority of cases of MRONJ are associated with antiresorptive agents including bisphosphonates, denosumab, and more recently romosozumab [4–7]. A notably smaller number of cases are associated with the use of antiangiogenic agents, both in individuals who also take antiresorptive drugs and in those who are antiresorptive drugs-naïve [8]. MRONJ can develop in approximately 7% of cancer patients taking high-potency bisphosphonates or high-dose denosumab and about 0.01–0.1% of those with osteoporosis using low-potency oral bisphosphonates or low-dosage denosumab [1, 9–12]. The use of antiangiogenic agents in combination with antiresorptive drugs is known to increase the risk of MRONJ development [13]; however, little is known regarding the incidence and prevalence of antiangiogenic-related MRONJ in antiresorptive drugs-naïve individuals. Antiangiogenic inhibitors have been increasingly used in the management of a range of malignancies including ovarian cancer, metastatic renal cell cancer, breast cancer, colorectal cancer, non-small-cell lung cancer (NSCLC), and glioblastoma multiforme [14]. Antiangiogenic inhibitors can be categorised into three major groups based on their mechanism of action: anti-VEGF monoclonal antibody (e.g., bevacizumab), VEGF decoy receptors or VEGF-Trap (e.g., aflibercept), and small molecule tyrosine kinase inhibitors (TKI) that block the VEGF receptors downstream signaling pathways (e.g., sunitinib, cabozantinib, and sorafenib) [15] (Table 1). Additionally, the mammalian target of rapamycin (mTOR) inhibitors also seems to have antiangiogenic effects by inhibiting the production of VEGF and platelet-derived growth factors (PDGF) [16–18].

The number of patients developing MRONJ associated with antiangiogenic inhibitors or a combination of antiangiogenics and antiresorptive drugs has been growing over the last few years [8, 13, 19, 20]. The purpose of the present study is to provide a comprehensive review of the published reports of MRONJ associated with antiangiogenic agents in patients with no history of antiresorptive therapies.

## 2. Materials and Methods

### 2.1. Literature Search Strategy

A literature search was conducted to identify clinical trials, case reports, and case series on MRONJ associated with antiangiogenic treatment in antiresorptive drugs-naïve individuals using MEDLINE via PubMed (up to December 2017), EMBASE (from 1980 to December 2017), and Web of Science (from 1900 to December 2017). The search strategy used the following keywords: “osteonecrosis,” “jaw osteonecrosis,” “jaw bone necrosis,” “oral osteonecrosis,” “antiangiogenic,” “angiogenesis inhibitors,” “antineoplastic agents,” “antiangiogenic activity,” “antiangiogenic therapy,” “chemotherapy,” and “targeted therapy.” The references of retrieved articles were manually searched in order to identify additional relevant articles and abstracts. The search included articles published in English and other languages. Inclusion criteria were patients developing MRONJ associated with antiangiogenic agents based on the definition of MRONJ proposed by the special committee on MRONJ of the American Association of Oral and Maxillofacial Surgeons (AAOMS) in 2014 [1]. Patients with history of radiotherapy involving the jaw bones and patients having previous history or concurrent use of antiresorptive therapy were excluded.

## 3. Results

### 3.1. Search Results

**Figure 1:**
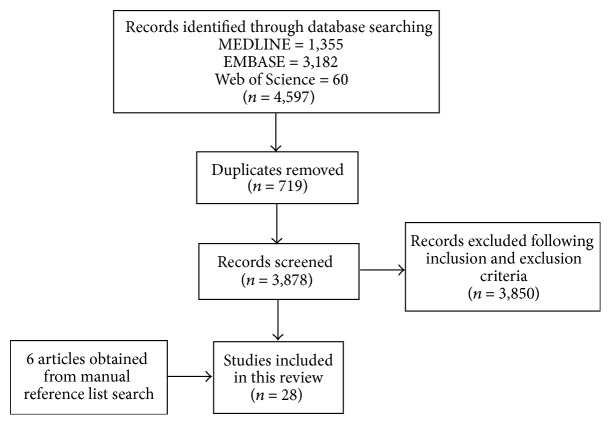
Flow chart of the study selection process.

A total of 4,597 articles were retrieved by the initial search, including literature reviews, duplicate articles, clinical trials, and case reports with bisphosphonates and antiangiogenic treatment. The flow chart of review process to identify studies included and excluded is shown in Figure 1. Following screening the articles, we identified 28 articles describing 35 cases of MRONJ meeting the aforementioned inclusion criteria. Of these 28 publications, 26 papers were published in English, one was published in Italian, and one was published in Japanese. These 35 reported MRONJ cases were related to previous history of treatment with bevacizumab (14 cases), aflibercept (5 cases), sunitinib (3 cases), cabozantinib (2 cases), sorafenib (1 case), temsirolimus (1 case), everolimus (1 case), dasatinib (1 case), and multiple antiangiogenic agents (7 cases) (Table 2).

All 35 patients were reported to have developed MRONJ associated with at least one antiangiogenic agent and without a history of treatment with antiresorptive drugs. There were 19 males (54.29%) and 14 males (40%). The mean age of patients was 59.06 years (range: 33–80 years). The underlying diseases that required treatment with antiangiogenic agents included metastatic renal cell cancer (10 patients, 28.57%) followed by metastatic colorectal cancer (6 patients, 17.14%), metastatic breast cancer (5 patients, 14.29%), and other cancers (14 patients, 40%).

The most common presenting symptom was pain to the mandible/maxilla (12 patients, 34.29%) whereas 8 individuals (22.86%) reported no notable symptoms. The remaining patients had a variety of presenting complaints including mild discomfort to the mandible (1 patient, 2.86%), spontaneous teeth loss (1 patient, 2.86%), gingival bleeding (1 patient, 2.86%), and limited mouth opening together with submandibular swelling (1 patient, 2.86%). Moreover, there were 6 patients (17.14%) presenting with multiple symptoms including pain to the jaw, halitosis, spontaneous tooth loss, ulceration, difficulty in chewing, and paraesthesia. Regarding clinical characteristics of MRONJ, 32 patients (91.43%) had intraoral frank bone exposure, while the other three patients had nonexposed MRONJ. Mandible was the most common area of MRONJ development (29 patients, 82.86%), whereas four patients (11.43%) developed MRONJ in the maxilla.

Fourteen patients (40%) were exposed to bevacizumab, followed by aflibercept (5 patients, 14.29%), sunitinib (3 patients, 8.57%), cabozantinib (2 patients, 5.71%), sorafenib (1 patient, 2.86%), temsirolimus (1 patient, 2.86%), everolimus (1 patient, 2.86%), dasatinib (1 patient, 2.86%), and multiple antiangiogenic agents (7 patients, 20%). Regarding the routes of drug administration, antiangiogenic medications were administered intravenously in 21 patients (60.00%), while 12 patients (34.29%) were given antiangiogenic therapy orally. Two patients (5.71%) were given the combination of intravenous administration and oral administration. The mean duration of intravenous and oral antiangiogenic therapy before MRONJ development was 6.49 months (range: 0.23–36; SD = 1.82; 95% CI: 2.67–10.30) and 16.72 months (range: 1–60; SD = 6.42; 95% CI: 2.59–30.84), respectively. Patients with MRONJ also received a variety of concomitant medications including chemotherapy, hormone therapy, corticosteroids, antihypertensive drugs, antidepressants, and gastrointestinal medications.

Additional risk factors for MRONJ were reported in 21 patients, with dental extraction being the most prominent factor (13 patients, 37.14%). Other factors included history of mucosal trauma from dentures, chronic infection/inflammation to the tooth-bearing alveolar bone (periodontal disease), and insertion of osteointegrated dental implants (8 patients, 22.86%). The mean time to MRONJ diagnosis after tooth extraction was 3.09 months (range: 0.23–8; SD = 1.13; 95% CI: 0.40–5.77).

Regarding the management of MRONJ, seventeen patients (48.57%) were managed with surgical procedures alone or combined with medications (antibiotic therapy, antimicrobial mouthwash) and interruption of antiangiogenic agents. 16 patients did not receive surgery (45.71%), with antiangiogenic agents being discontinued in 7 cases. There was no active intervention reported in one patient. Most surgical interventions (11 patients) were minimally invasive procedures including smoothening of exposed bone, local flap coverage, removal of superficial necrotic bone, soft tissue debridement, and bone curettage, whereas 6 patients underwent major surgery such as bone decortication, resection of necrotic bone with local flap coverage, segmental osteotomy, and block resection. The outcomes of therapy were reported for 29 patients (82.8%), whereas no information was provided for the other 6 cases. 18 patients out of these 29 (62%) were reported to have disease resolution, while 11 patients showed persistent bone exposure. Disease resolution was described as complete mucosal coverage/no evidence of exposed bone in 13 cases, whereas no clear description was provided for the remaining 5 cases. Of note, one patient who experienced disease resolution to the left side of the mandible eventually developed a new area of MRONJ to the right mandible. The mean time from MRONJ diagnosis to complete healing was 6.75 months (range months: 1.84–22; SD = 2.47; 95% CI: 0.90–12.59). The data of 35 reported cases with MRONJ associated with antiangiogenics are summarised in Table 3.

## 4. Discussion

The present study is the first comprehensive review upon MRONJ in patients treated with antiangiogenics in the absence of bone-modulating therapy.

We present data from 35 patients with different metastatic cancers who developed MRONJ following antiangiogenic treatments. All individuals were antiresorptive drugs-naïve. We have identified a number of differences between MRONJ associated with antiangiogenic agents and MRONJ associated with antiresorptive drugs. Our data showed a sex ratio of 4 : 5 (F : M) and an age range of 33–80 years (mean: 59.06 years), compared to sex ratio of 3 : 2 and age range of 42–90 years (mean 66 years) reported for antiresorptive drugs-associated MRONJ [50–52]. There also seem to be differences in the prevalence of MRONJ in these two populations. The reported prevalence of MRONJ in patients who had been treated with intravenous bevacizumab alone for the treatment of advanced breast cancer was 0.2%, which was lower than that of MRONJ associated with intravenous antiresorptive agents (7%) [11, 19]. However, it is important to note that the prevalence of MRONJ related to antiangiogenic agents may also depend on the epidemiology of underlying malignancies that require antiangiogenic therapy.

The clinical presentations of MRONJ associated with antiangiogenics also seem to be different from MRONJ due to antiresorptive agents. Approximately up to 25% of MRONJ cases related to antiresorptive medications can present without frank bone exposure [53], whereas most of the patients in the present review had clear evidence of bone exposure (91.43%). However, the number of patients with nonexposed MRONJ might be underestimated, since until 2014 MRONJ could only be diagnosed in individuals with clinical evidence of exposed bone as per AAOMS definition [53, 54].

With respect to the presenting complaints and location, they appear to be similar in two populations. The majority of patients with antiangiogenic-related MRONJ in this study experienced pain to the jaw, which is also the most common complaint in patients with MRONJ associated with antiresorptive agents [51, 55]. In the present study, most MRONJ cases associated with antiangiogenic therapy tended to occur in the mandible more frequently than in the maxilla, similar to those with antiresorptive drugs-induced MRONJ [50, 52].

A number of additional risk factors were identified in the present review including dental extraction, the use of denture, periodontal infection, and dental implant. Almost 40% of reported cases in this study were predominantly preceded by tooth extraction, which is similar to those with antiresorptive drugs-related MRONJ [50, 56].

There is a slight difference with respect to underlying malignancies between two populations. Patients with antiresorptive drugs-associated MRONJ showed the previous history of multiple myeloma, metastatic breast cancer, and metastatic prostate cancer, whereas those with MRONJ related to antiangiogenic medications in our review were mainly diagnosed with metastatic renal cell cancer, followed by metastatic colorectal cancer and metastatic breast cancer as demonstrated in Table 3 [52].

Although there was no consistent pattern in the time to MRONJ development in this review, the average time for developing MRONJ among patients with either intravenous or oral antiangiogenics was shorter than the average time to MRONJ onset in those treated with antiresorptive drugs. The mean time to event for intravenous and oral antiangiogenic agents in this study was 6.5 and 16.71 months, respectively, while it was reported to be approximately 1.8 and 3 years for bisphosphonate therapy [12, 57].

Patients with metastatic malignancy may receive a number of anticancer drugs simultaneously. In this review, we found that seven of the reported cases received more than one antiangiogenic agent in their treatment history [32, 44–49]. Of these patients, some were given different antiangiogenics at the same time, while others received these agents at different time points. The development of MRONJ is usually associated with the latest antiangiogenic agent used by the patient; however, one cannot exclude the fact that the antiangiogenic agents previously used by these patients might have contributed to it.

We included in this review two cases of MRONJ associated with new TKIs, namely, pazopanib in combination with axitinib (*n* = 1) and dasatinib (*n* = 1) [32, 49]. According to the Food and Drug Administration's Adverse Event Reporting System (FAERS), pazopanib and axitinib have been associated with the development of MRONJ in 10 and 9 individuals, respectively; however, as data regarding concurrent or previous medication were not available in FAERS documentation [58], it is difficult to conclude whether these individuals were indeed antiresorptive drugs-naïve. Therefore, we decided not to include these 19 cases in our review.

With regard to the management of MRONJ, approximately half of the individuals with MRONJ associated with antiangiogenics (48.57%) were managed surgically, which is similar to those with bisphosphonate-related MRONJ [11, 52]. However, the prognosis of antiangiogenic-related MRONJ appears to be better than that of individuals developing MRONJ associated with antiresorptive agents. We observed a 62% rate of disease resolution in those where outcomes were reported as opposed to approximately 50% reported in the literature for MRONJ associated with antiresorptive agents [56, 59, 60]. It is possible that the higher rate of disease resolution might be related to the shorter half-life of antiangiogenics [61, 62], as well as the lower cumulative dosages [63]. Moreover, the average time to resolution for MRONJ associated with antiangiogenics appears to be shorter than antiresorptive drugs-induced MRONJ (6.75 months, range: 1.84–22 months versus 8.2 months, range: 0.2–25.6 months) [55].

In this comprehensive review, we excluded a number of potential antiangiogenic-related MRONJ cases due to a lack of adequate clinical information. For example, the 2012 report on aflibercept by the US Food and Drug Administration (FDA) described 3 aflibercept-treated bisphosphonate-naïve patients who developed MRONJ; however, none of these patients were added to the present review as one had jaw bone exposure for less than 8 weeks and no information was provided for the other two cases [64]. Furthermore, in a pivotal BOLERO-2 trial, MRONJ has been described in 2 patients in the experimental arm (everolimus-exemestane) and 1 patient in the control arm (exemestane), with one of three patients to receive bisphosphonate treatment [65]. However, there was no evidence to show whether the patient with a history of bisphosphonate treatment was in the experimental arm or control arm. More recently, Antonuzzo et al. [66] reported the first case with MRONJ potentially associated with regorafenib, one of the tyrosine kinase inhibitors, in an antiresorptive drugs-naïve individual. Although MRONJ appeared 22 months after regorafenib treatment, Fusco et al. [67] have noted that some details such as the use of other medications prior to regorafenib treatment, dosing, and the time on medication are still missing. This medication is usually used as a third or further line of treatment of metastatic colorectal cancer. Therefore, it is also worth knowing whether this patient has received other well-documented antiangiogenic medications such as bevacizumab and aflibercept prior to regorafenib. If this is the case, bevacizumab or aflibercept possibly might contribute to the development of MRONJ rather than regorafenib alone. Another patient with gastrointestinal stromal tumours (GISTs) receiving imatinib monotherapy presented with pain and exposed bone at lower right mandible after having the tooth removed for 5 weeks. The patient was treated with debridement of necrotic bone and antibiotic and then was discharged. Unfortunately, there was no further information about this patient [68]. The duration of persistent bone exposure in this case was not mentioned if it was longer than 8 weeks. Therefore, available data seems not to be enough to classify this case as MRONJ according to the definition of MRONJ [1] and to confirm the association between MRONJ and imatinib. In addition to the above reported cases, Hopp et al. [69] reported one patient with necrotic bone exposure after the 2-year intravitreal injections of bevacizumab for treatment of retinal vascular thrombosis without notable dental risk factors or use of bisphosphonates. After the patient experienced pain to the mandible, the lesion was completely healed by antibiotics treatment within 8 weeks. Therefore, this case seems not to be correlated with the definition of MRONJ formulated by the AAOMS in 2014 [1].

It is important to highlight that another case of oral soft tissues complication associated with bevacizumab was also reported by Magremanne et al. [70]. Although this case was included in previous reviews regarding cases of MRONJ associated with antiangiogenic agents, there was no evidence of osteonecrosis of the jaw and also the necrotic area seemed to be limited only to oral soft tissues. This reported case does not meet the definition of MRONJ and hence it was excluded from this review.

## 5. Conclusion

There remains incomplete information regarding the cases of antiangiogenic-related MRONJ in antiresorptive-naïve individuals reported in the literature. It is therefore difficult to draw any conclusion regarding the epidemiology and the characteristics of MRONJ in this patient population. Within the limitation of available data, we have identified a number of differences between MRONJ associated with antiangiogenics and MRONJ related to antiresorptive drugs including demographic characteristics, prevalence, the underlying malignant disease, time to the onset, and time to resolution. Considering that the list of antiangiogenic inhibitors that have potential to increase the risk of MRONJ development is increasing, further prospective and well-designed research is warranted to confirm our findings and increase knowledge and understanding of the disease.

## Figures and Tables

**Table 1:** Approved antiangiogenic medications [14, 15, 21, 22].

Approved antiangiogenic drugs	
*Anti-VEGF monoclonal antibody*	*Indications for use*
Bevacizumab	Metastatic colorectal cancer
	Non-small-cell lung cancer
	Glioblastoma multiforme
	Metastatic renal cell cancer
	Macular degeneration
	Metastatic HER2 negative breast cancer
	Persistent, recurrent, and metastatic cervical cancer
	Platinum-resistant recurrent epithelial ovarian, fallopian tube or primary peritoneal cancer

*VEGF decoy receptor (VEGF-Trap)*	*Indications for use*
Aflibercept	Metastatic colorectal cancer

*Tyrosine kinase inhibitors*	*Indications for use*
Sorafenib	Metastatic renal cell cancer
	Hepatic cancer (hepatocellular carcinoma)
Sunitinib	Metastatic renal cell carcinoma
	Gastrointestinal stromal tumour
	Pancreatic neuroendocrine tumour
Cabozantinib	Medullary thyroid cancer
Erlotinib	Non-small-cell lung cancer
	Pancreatic cancer
Axitinib	Metastatic renal cell cancer
Pegaptanib	Macular degeneration
Ranibizumab	Macular degeneration
Pazopanib	Metastatic renal cell cancer
	Soft tissue sarcoma
Vandetanib	Medullary thyroid cancer
Regorafenib	Metastatic colorectal cancer
	Gastrointestinal stromal tumour
Imatinib	Chronic myeloid leukemia
	Renal cell cancer
	Gastrointestinal stromal tumour
Dasatinib	Philadelphia chromosome-positive (Ph+) chronic
	myeloid leukemia (CML)
	Chronic phase Ph+ CML
	Philadelphia chromosome-positive (Ph+) acute lymphoblastic leukemia (Ph+ ALL)

*Mammalian target of rapamycin inhibitors (mTOR inhibitors)*	*Indications for use*
Temsirolimus	Renal cell cancer
Everolimus	Advanced breast cancer
	Advanced renal cell cancer
	Pancreatic neuroendocrine tumour
	Tuberous sclerosis complex
	Subependymal giant cell astrocytoma

**Table 2:** Previously reported cases of MRONJ associated with antiangiogenic medications (*n* = 35).

Number	Authors	Age	Sex	Diagnosis of cancer	Treatment and concurrent medications	Antiangiogenic agents	Symptoms	Clinical presentation	Site of MRONJ	Time to MRONJ	Predisposing factors	Management of MRONJ	Outcomes	Definition of disease resolution
(1)	Estilo et al. [23]	51	F	Metastatic breast cancer	Mastectomy Chest wall resection Chest wall radiation Doxorubicin Cyclophosphamide Letrozole Paclitaxel Capecitabine	Bevacizumab 15 mg/kg every 3 weeks (total 8 doses)	Jaw discomfort	Bone exposure	Mandible	18 weeks after starting bevacizumab	None	Surgical treatment (smoothen exposed bone) Chlorhexidine mouthwash 0.12% Interruption of bevacizumab	Disease resolution (few weeks) Developed new MRONJ lesion (right mandible)	Complete mucosal coverage

(2)	Estilo et al. [23]	33	F	Glioblastoma multiforme	Surgical resection Radiotherapy Temozolomide	Bevacizumab 15 mg/kg every 2 weeks	Jaw pain (gingival pain)	Bone exposure	Mandible	11 weeks after starting bevacizumab	None	None	Persistent bone exposure (3 months)	—

(3)	Greuter et al. [24]	63	F	Metastatic breast cancer	Liposomal-doxorubicin	Bevacizumab	Maxillary pain	Bone exposure	Maxilla	2 months after starting bevacizumab	Extraction due to dental infection (1 month)	Surgical treatment	Disease resolution	Not specified

(4)	Serra et al. [25]	64	M	Metastatic non-small-cell lung cancer	Pneumectomy Lymph node ablation Cisplatin Gemcitabine	Bevacizumab 7.5 mg/kg	Jaw pain	Bone exposure	Mandible	1 week after starting bevacizumab	Extraction (1 week)	Surgical treatment (local flap coverage) Amoxicillin with clavulanate Chlorhexidine mouthwash 0.2%	Persistent bone exposure (3.5 months)	—

(5)	Guarneri et al. [19]	NA	NA	Metastatic breast cancer	Docetaxel	Bevacizumab 7.5 mg/kg or 15 mg/kg every 3 weeks	NA	Bone exposure	Mandible	7 months after starting bevacizumab	None	Surgical treatment (mandible decortication, tooth extraction) Interruption of bevacizumab	Disease resolution (6 months)	Complete mucosal coverage

(6)	Guarneri et al. [19]	NA	NA	Metastatic breast cancer	Docetaxel	Bevacizumab 7.5 mg/kg or 15 mg/kg every 3 weeks	NA	Bone exposure	NA	2 months after starting bevacizumab	None	NA	NA	—

(7)	Brunamonti Binello et al. [26]	47	M	Adenocarcinoma of parotid gland	Surgical treatment Epirubicin Cisplatin	Bevacizumab 15 mg/kg (total 8 doses in 6 months)	Jaw pain, paraesthesia	Bone exposure	Mandible	16 months after starting bevacizumab	Symptomatic eruption of lower third molar	Surgical treatment (removed necrotic bone) Amoxicillin with clavulanate Metronidazole	Persistent bone exposure (7 months)	—

(8)	Bettini et al. [27]	57	F	Metastatic non-small-cell lung cancer	Gemcitabine Cisplatin Corticosteroid	Bevacizumab 945 mg/21 days 4 cycles	Jaw pain, halitosis, tooth loss	Bone exposure	Mandible	2 months after starting bevacizumab	Periodontal infection	Amoxicillin with clavulanate Lincomycin (for 7 days)	Disease resolution	Complete mucosal coverage

(9)	Dişel et al. [28]	51	M	Metastatic colon cancer	5-Fluorouracil Leucovorin Oxaliplatin	Bevacizumab 5 mg/kg every 2 weeks	Jaw pain, ulcer, difficulty in chewing	Bone exposure	Mandible	NA	None	Surgical treatment (bone curettage)	NA	—

(10)	Sato et al. [29]	67	M	Metastatic sigmoid colon cancer	Surgical treatment Oxaliplatin Leucovorin Irinotecan 5-Fluorouracil	Bevacizumab	Jaw pain	Nonexposed MRONJ	Maxilla	3 months after starting bevacizumab	Extraction (1 month)	Surgical treatment (removal of necrotic tissue) Antibiotics	Disease resolution	Complete mucosal coverage

(11)	Fusco et al. [30]	60	M	Metastatic rectal cancer	Surgical treatment Radiotherapy 5-Fluorouracil Leucovorin Irinotecan Oxaliplatin	Bevacizumab	Jaw pain	Bone exposure	Mandible	9 months after starting bevacizumab	Extraction (8 months)	Antibiotics Chlorhexidine mouthwash	NA	—

(12)	Tzermpos et al. [31]	69	M	Metastatic non-small-cell lung cancer	Carboplatin Docetaxel Cortisone	Bevacizumab 15 mg/kg every 3 weeks	Jaw pain, discomfort, paraesthesia	Bone exposure	Mandible	3 years after starting bevacizumab	Denture	Surgical treatment (surgical debridement) Amoxicillin Metronidazole Chlorhexidine mouthwash 0.12% Interruption of bevacizumab	Disease resolution (8 weeks)	Complete mucosal coverage

(13)	Abel Mahedi Mohamed et al. [32]	55	F	Non-small-cell lung cancer	Corticosteroids	Bevacizumab	Asymptomatic	Bone exposure	Maxilla	1.5 months after starting bevacizumab	Extraction	Conservative treatment	Disease resolution	Not specified

(14)	Abel Mahedi Mohamed et al. [32]	66	M	Glioblastoma multiforme	Corticosteroids	Bevacizumab	Pain	Nonexposed MRONJ	Mandible	1.5 months after starting bevacizumab	Trauma	Conservative treatment (antibiotic treatment)	Disease resolution	Not specified

(15)	Ponzetti et al. [33]	64	F	Metastatic colorectal cancer	5-Fluorouracil Irinotecan	Aflibercept	Spontaneous teeth loss with purulent discharge	Bone exposure	Mandible	22 weeks after starting aflibercept	Periodontal infection	Laser treatment	Persistent bone exposure	—

(16)	Mawardi et al. [34]	43	M	Metastatic colorectal cancer	5-Fluorouracil Leucovorin Irinotecan	Aflibercept	Jaw pain	Bone exposure	Mandible	32 weeks after starting aflibercept	None	Amoxicillin Chlorhexidine mouthwash	Persistent bone exposure (1.5 months)	—

(17)	Mawardi et al. [34]	63	M	Metastatic carcinoid cancer	NA	Aflibercept	Asymptomatic	Bone exposure	Mandible	46 weeks after starting aflibercept	None	Amoxicillin with clavulanate Chlorhexidine mouthwash	Persistent bone exposure (2.5 months)	—

(18)	Mawardi et al. [34]	51	M	Metastatic esophageal cancer	5-Fluorouracil Leucovorin Oxaliplatin	Aflibercept	Jaw pain	Bone exposure	Mandible	14 weeks after starting aflibercept	Extraction (2 weeks)	Amoxicillin Chlorhexidine mouthwash Nonsurgical sequestrectomy	Persistent bone exposure (2 months)	-

(19)	Zarringhalam et al. [35]	47	M	Metastatic colorectal, peritoneum, liver, and pelvic cancer	None	Aflibercept	Asymptomatic	Bone exposure	Mandible	4 weeks after starting aflibercept	None	Surgical treatment (smoothen sharp exposed bone)	Persistent bone exposure (12 weeks)	—

(20)	Nicolatou-Galitis et al. [36]	64	F	Metastatic renal cell cancer	Nephrectomy T4 replacement therapy Prednisolone (50 mg/day)	Sunitinib 50 mg/day for 4 weeks on and 2 weeks off (for 4 years)	Jaw pain	Bone exposure	Mandible	4 years after starting sunitinib	Denture	Amoxicillin Chlorhexidine mouthwash Azithromycin Interruption of sunitinib	Disease resolution (3 months)	Complete mucosal coverage

(21)	Fleissig et al. [37]	58	F	Metastatic renal cell cancer	Nephrectomy Thyroxin sodium	Sunitinib 50 mg/day for 4 weeks on and 2 weeks off	Limited mouth opening, submandibular swelling, pain	Bone exposure	Mandible	10 months after starting sunitinib	Extraction (8 months)	Amoxicillin with clavulanate (IV) PenG (IV) for 6 weeks and oral amoxicillin for 6 weeks Interruption of sunitinib	Disease resolution (18 weeks)	Complete mucosal coverage

(22)	Melloni et al. [38]	62	M	Metastatic renal cell cancer	NA	Sunitinib 50 mg/day for 4 weeks on and 2 weeks off	Jaw pain and infected lesion to the cutaneous side of the jaw	Bone exposure	Mandible	5 years after starting sunitinib	None	Surgical treatment (surgical sequestrectomy, ablation of necrotic bone, and local flap coverage) Amoxicillin with clavulanate Ofloxacin Interruption of sunitinib	Disease resolution (12 months)	Complete mucosal coverage

(23)	Tempia Valenta et al. [39]	51	F	Medullary thyroid cancer	NA	Cabozantinib	NA	Bone exposure	Mandible	6 months after starting cabozantinib	Extraction	Surgical treatment (surgical debridement) Amoxicillin and clavulanate Chlorhexidine mouthwash 0.2%	Disease resolution (22 months)	Not specified

(24)	Marino et al. [40]	51	F	Medullary thyroid cancer	Thyroidectomy 5-Fluorouracil Dacarbazine Radiotherapy Levothyroxine Calcitriol Vitamin D3 Duloxetine Propranolol Lansoprazole Loperamide	Cabozantinib (175 mg/day)	Asymptomatic	Bone exposure	Mandible	3 months after starting cabozantinib	Extraction due to dental infection (3 months)	Surgical treatment (segmental ostectomy and tooth extraction) Amoxicillin and clavulanate Chlorhexidine mouthwash 0.2%	Disease resolution	Complete mucosal coverage

(25)	Garuti et al. [41]	74	M	Metastatic hepatocellular carcinoma	Furosemide Potassium canrenoate Bisoprolol Allopurinol Tamsulosin Hydroxychloroquine Vitamin D Sertraline	Sorafenib 400 mg/day	Asymptomatic	Nonexposed MRONJ	Mandible	3 months after starting sorafenib	None	Interruption of sorafenib	Persistent bone exposure (3 months)	—

(26)	Abel Mahedi Mohamed et al. [32]	53	F	Acute lymphoblastic leukemia	Corticosteroids	Dasatinib	Pain	Bone exposure	Mandible	5 months after starting dasatinib	Extraction	Surgical treatment (block resection)	Disease resolution	Not specified

(27)	Parti et al. [42]	60	M	Metastatic renal cell cancer	Nephrectomy Prostatectomy	Temsirolimus 25 mg every week	NA	Bone exposure	Mandible	3 months after starting temsirolimus	Extraction (3 months)	Interruption of temsirolimus	NA	—

(28)	Yamamoto et al. [43]	80	F	Metastatic breast cancer	Capecitabine Tamoxifen Fulvestrant Exemestane	Everolimus	Jaw pain, localised heat, tenderness	Bone exposure	Mandible	2 months after starting everolimus	None	Interruption of everolimus	Persistent bone exposure (2 months)	—

(29)	Agostino et al. [44]	73	M	Metastatic renal cell cancer	Nephrectomy	(1) Sunitinib 50 mg/day for 4 weeks of 6-week cycle (2) Temsirolimus 25 mg every week (3) Bevacizumab 10 mg/kg every two weeks	NA	NA	NA	12 months after starting bevacizumab	NA	Interruption of bevacizumab	NA	—

(30)	Koch et al. [45]	59	M	Metastatic renal cell cancer	Nephrectomy Interferon Vinblastine Ramipril Hydrochlorothiazide Metoprolol I-Thyroxin	(1) Sorafenib (2) Sunitinib 50 mg/day for 4 weeks and then sunitinib 37.5 mg/day	Asymptomatic	Bone exposure	Mandible	51 months after starting sunitinib	Extraction (2 months)	Surgical treatment (ablation of necrosis and local flap coverage)	Disease resolution	Complete mucosal coverage

(31)	Santos-Silva et al. [46]	61	M	Metastatic renal cell cancer	Nephrectomy Hydrochlorothiazide Captopril	(1) Bevacizumab 10 mg/kg every 2 weeks (2) Temsirolimus 25 mg every week	Jaw pain	Bone exposure	Mandible	55 weeks after starting bevacizumab and temsirolimus	None	Chlorhexidine mouthwash 0.12% Interruption of bevacizumab and temsirolimus	Disease resolution (3 months)	The absence of exposed necrotic bone

(32)	Pakosch et al. [47]	53	F	Pancreatic cancer	Surgical treatment Gemcitabine Leucovorin 5-Fluorouracil Oxaliplatin Paclitaxel Erlotinib	(1) Bevacizumab (2) Sorafenib	Jaw pain	Bone exposure	Mandible	4 months after starting bevacizumab and sorafenib	Denture	Surgical treatment (decortication), Amoxicillin with clavulanate Chlorhexidine mouthwash Solcoseryl Interruption of bevacizumab and chemotherapy	Disease resolution (2 months)	Complete mucosal coverage

(33)	Jung [48]	62	F	Renal cell cancer	Nephrectomy	(1) Pazopanib (2) Everolimus	Gingival bleeding and sore gum	Bone exposure	Mandible	7 weeks after starting everolimus	Dental implant	Cephalosporin Surgical treatment (sequestrectomy and internal fixation)	Disease resolution	Complete mucosal coverage

(34)	Patel et al. [49]	67	M	Metastatic renal cell cancer	Nivolumab Amlodipine Ramipril Levetiracetam Dexamethasone Lansoprazole Morphine Metoclopramide Amiodarone Cholecalciferol	(1) Pazopanib (2) Axitinib	Asymptomatic	Bone exposure	Maxilla	1 months after starting axitinib	None	Hydrogen peroxide mouthwash	NA	—

(35)	Abel Mahedi Mohamed et al. [32]	70	M	Renal cell cancer	Corticosteroids	(1) Sunitinib (2) Everolimus	Asymptomatic	Bone exposure	Mandible	10 months after starting sunitinib, everolimus was commenced	Extraction	Conservative treatment	Persistent bone exposure	—

NA: not available.

**Table 3:** Summary of data of reported cases of antiangiogenic-related MRONJ (*n* = 35).

Age (years, range)	
Mean	59.06 (33–80)
Gender (*n*, %)	
Male	19 (54.29%)
Female	14 (40.00%)
NA	2 (5.71%)
Diagnosis of cancers (*n*, %)	
Metastatic renal cell cancer	10 (28.57%)
Metastatic colorectal cancer	6 (17.14%)
Metastatic breast cancer	5 (14.29%)
Other cancers	14 (40.00%)
Metastatic non-small-cell lung cancer	4
Glioblastoma multiforme	2
Medullary thyroid cancer	2
Malignant parotid tumour	1
Pancreatic cancer	1
Metastatic hepatocellular carcinoma	1
Metastatic carcinoid cancer	1
Metastatic oesophageal cancer	1
Presenting complaints (*n*, %)	
Jaw pain	12 (34.29%)
Jaw pain with other complaints	6 (17.14%)
Asymptomatic	8 (22.86%)
Jaw discomfort	1 (2.86%)
Spontaneous teeth loss	1 (2.86%)
Limited mouth opening and submandibular area swelling	1 (2.86%)
Gingival bleeding	1 (2.86%)
NA	5 (14.29%)
Clinical presentation (*n*, %)	
Bone exposure MRONJ	32 (91.43%)
Nonexposed MRONJ	3 (8.57%)
Location	
Mandible	29 (82.86%)
Maxilla	4 (11.43%)
NA	2 (6.67%)
Types of antiangiogenic agents (*n*, %)	
Bevacizumab	14 (40%)
Aflibercept	5 (14.29%)
Sunitinib	3 (8.57%)
Cabozantinib	2 (5.71%)
Sorafenib	1 (2.86%)
Temsirolimus	1 (2.86%)
Everolimus	1 (2.86%)
Dasatinib	1 (2.86%)
Multiple antiangiogenic agents	7 (20.00%)
Route of antiangiogenic administrations (*n*, %)	
Intravenous administration	21 (60.00%)
Oral administration	12 (34.29%)
Combination of intravenous administration and oral administration	2 (5.71%)
Time to MRONJ (months, 95% CI)	
Intravenous antiangiogenics	6.49 (2.67–10.30)
Oral antiangiogenics	16.72 (2.59–30.84)
Predisposing factors (*n*, %)	
Extraction	13 (37.14%)
Periodontal disease	3 (8.57%)
Minor trauma from use of denture	4 (11.43%)
Dental implant	1 (2.86%)
Mean time to MRONJ after extraction (months, 95% CI)	3.09 (0.40–5.77)
Management of MRONJ (*n*, %)	
Surgical treatment	17 (48.57%)
Minimally invasive surgical procedures	11
Major surgical procedures	6
Nonsurgical treatment	16 (45.71%)
No treatment	1 (2.86%)
NA	1 (2.86%)
Treatment outcomes (*n*, %)	
Disease resolution	18 (62.06%)
Mean time to resolution (months, 95% CI)	6.75 (0.90–12.59)
Incomplete resolution	11 (37.93%)
NA	6

NA: not available.
